# DRESS syndrome: à propos de trois observations

**DOI:** 10.11604/pamj.2014.19.166.4648

**Published:** 2014-10-17

**Authors:** Wafa Chebbi, Jihed Souissi, Jihène Chelli, Fatma Larbi, Baha Zantour, Mohamed Habib Sfar

**Affiliations:** 1 Service de Médecine Interne, CHU Taher Sfar Mahdia, 5100 Mahdia, Tunisie

**Keywords:** Syndrome d′hypersensibilité médicamenteuse, carbamazipine, salozopyrine, DRESS syndrome, carbamazipine, salozopyrine

## Abstract

Le syndrome d'hypersensibilité médicamenteuse ou Drug Rash with Eosinophilia and Systemic Symptoms (DRESS) est une toxidermie rare mais sévère. Nous rapportons trois observations de DRESS syndromes secondaires à la prise de carbamazipine dans deux cas et de salazopyrine dans un cas. Le délai moyen entre la prise médicamenteuse et la survenue du DRESS syndrome était de six semaines. Le médicament incriminé était arrêté d'une façon définitive dans tous les cas. Une corticothérapie par voie générale était instaurée chez tous les patients devant l'atteinte hépatique sévère. L’évolution était favorable avec disparation des lésions cutanées et normalisation du bilan hépatique. Le diagnostic du syndrome DRESS doit être évoqué devant un tableau associant une éruption fébrile et des signes systémiques faisant suite à une prise médicamenteuse. La précocité du diagnostic est fondamentale pour l'arrêt définitif des médicaments suspects. Le traitement n'est pas bien codifié mais repose actuellement sur la corticothérapie générale.

## Introduction

Le syndrome d'hypersensibilité médicamenteuse ou Drug Rash with Eosinophilia and Systemic Symptoms (DRESS) est une toxidermie rare mais sévère [1]. Sa gravité est liée aux manifestations systémiques pouvant évoluer vers une défaillance multiviscérale et mettre en jeu le pronostic vital. La mortalité est estimée à 10%. Sa physiopathologie est aujourd'hui mieux comprise faisant intervenir un terrain immunogénétique prédisposant et une réactivation des herpès virus dominés par le virus HHV-6 [2]. Nous discutons, à travers trois observations, les caractéristiques cliniques, évolutives et thérapeutiques de DRESS syndrome et nous soulignons l'intérêt de diagnostic rapide et de traitement urgent de ce syndrome.

## Patient et observation

### Observation 1

**Figure 1:**
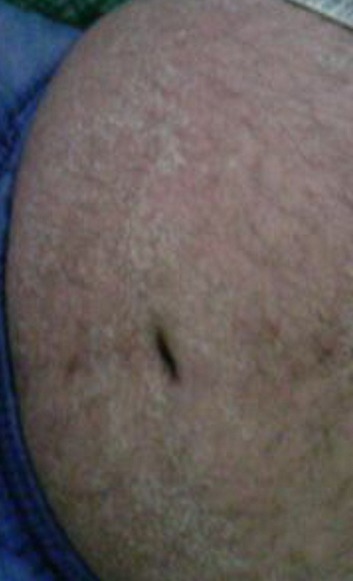
Éruption érythémateuse, maculopapuleuse et desquamative de l'abdomen

**Figure 2:**
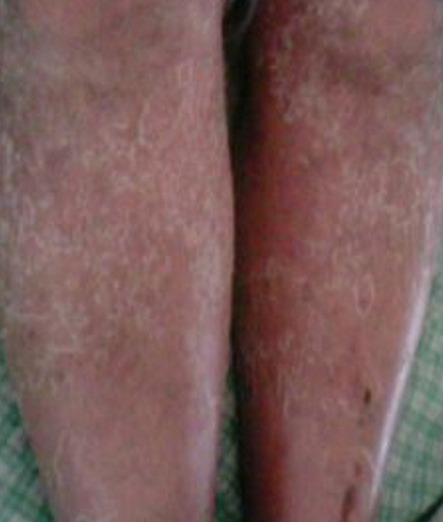
Éruption érythémateuse, maculopapuleuse et desquamative des membres inférieurs

Un homme âgé de 37ans était hospitalisé pour une éruption cutanée généralisée. Dans ses antécédents, il est suivi en psychiatrie pour un trouble bipolaire et traité par carbamazepine (Tégrétol^®^) et amisulpride (solian^®^) depuis 2 mois. L'histoire de sa maladie remonte à cinq jours avant son hospitalisation, marquée par l'installation brutale des douleurs abdominales avec des vomissements post-prandiaux associés à une fièvre et des frissons, suivis au bout de 2 jours, de l'apparition d'une éruption cutanée généralisée et prurigineuse. L'examen physique trouvait un patient fébrile à 39°C, un état général altéré, un œdème facial à prédominance péri-orbitaire, une éruption généralisée, érythémateuse, maculopapuleuse et desquamative (Figure 1, Figure 2) et des adénopathies axillaires et inguinales bilatérales de 2 cm de diamètre. L'examen abdominal était normal. Le bilan biologique montrait une hyperleucocytose à 18000 éléments/mm3, une hyperéosinophilie à 3180 éléments/ mm3, une cytolyse hépatique avec cholestase (ASAT 7 fois la normale, ALAT à 8 fois la normale, gamma glutamyl transférases (GT) 4 x normale, phosphatases alcalines 1,5 fois la normale) et un taux élèvé des lactates déshydrogénases (LDH) à 757 UI/l (normal, entre 200 et 450). Le taux de prothrombine était à 90%. Il y avait également un syndrome inflammatoire avec une CRP à 82 mg/L (normale < 5) et une vitesse de sédimentation à 100 mm/h. L′amylasémie et l′amylasurie étaient normales. La fonction rénale était conservée. La protéinurie de 24 h était négative. La radiographie pulmonaire et l’échographie abdominale étaient sans anomalies. Le bilan infectieux (sérologies du virus d'Epstein-Barr, cytomégalovirus, virus des hépatites A, B et C, virus de l'immunodéficience humaine (VIH), parvovirus B19 et hémocultures) revenait négatif. L'examen histologique d'une biopsie cutanée montrait un infiltrat inflammatoire dermique polymorphe associé à un œdème dermique sans nécrose, compatible avec une toxidermie. Le diagnostic de DRESS syndrome était retenu et la carbamazipine était immédiatement et définitivement interrompue. Une corticothérapie à la dose de 1 mg/kg/j était instaurée. L’évolution était favorable avec disparition de la fièvre, des adénopathies et régression complète des lésions cutanées au bout de deux semaines. Le bilan hépatique et le taux des éosinophiles étaient normalisés au bout de 4 semaines. Les tests épicutanés réalisés ultérieurement au centre de pharmacovigilance ont révélé une forte positivité à la carbamazipine. Aucune récidive n’était observée après un recul de 2 ans.

### Observation 2

Patiente âgée de 45 ans, aux antécédents de rectocolite hémorragique, était hospitalisée pour une éruption cutanée généralisée survenue six semaines après le début d'un traitement par salazopyrine. L'examen clinique trouvait une patiente fébrile à 40°C, un état général altéré, un œdème du visage, une éruption cutanée maculo-papuleuse, confluente par endroit et des adénopathies cervicales et axillaires de 1,5 cm de diamètre. A la biologie, il y avait une hyperéosinophilie à 2800 éléments/mm3 et une cytolyse hépatique avec transaminases 8 fois la normale. Les prélèvements bactériens (hémocultures, ECBU, prélèvements cutanés) et les sérologies virales (hépatites A, B et C, VIH, virus Epstein-Barr, cytomégalovirus et parvovirus B19) étaient négatifs. La radiographie thoracique était sans anomalies. La biopsie cutanée montrait une spongiose basale avec exocytose de lymphocytes et quelques polynucléaires éosinophiles et une discrète nécrose kératinocytaire. Le derme superficiel est œdémateux comportant un infiltrat inflammatoire fait de cellules mononuclées et quelques polynucléaires éosinophiles. Cet ensemble lésionnel dermo-épidermique était compatible avec une toxidermie médicamenteuse. Le diagnostic de DRESS syndrome était retenu. La salazopyrine était arrêtée et une corticothérapie générale à la dose de 1 mg/kg/j était instaurée. L’évolution était favorable avec disparition de la fièvre et régression complète des lésions cutanées au bout de dix jours. Le bilan hépatique et le taux des éosinophiles étaient normalisés au bout de 4 semaines. Aucune récidive n’était observée après un recul de 2 ans.

### Observation 3

Patiente âgée de 17 ans était hospitalisée pour une éruption cutanée prurigineuse généralisée avec fièvre et frissons. Dans ses antécédents, elle est suivie pour une épilepsie révélée tardivement à l’âge de huit ans par un état de mal convulsif. Le bilan étiologique de cette épilepsie était négatif et un traitement par l′acide valproïque (Dépakine^®^) était mis en route. Un mois avant son hospitalisation, les crises convulsives s’étant majorées, la carbamazépine (Tégrétol^®^) a été introduite à dose progressive. A l'examen, la patiente était fébrile à 40°C, la pression artérielle était à 130/ 80 mmHg, le pouls à 120 battements par minute. Sur le plan cutané, il existait une éruption maculopapuleuse généralisée, les paumes et les plantes étaient atteintes et il n'y avait pas d'atteinte muqueuse. Il y avait des adénopathies sous-mandibulaires, occipitales et axillaires centimétriques. La numération formule sanguine montrait une hyperleucocytose à 20500/mm3 avec des éosinophiles à 3600 éléments/mm3 et des monocytes à 2800/mm3. Le bilan hépatique montrait une activité de l'ALAT à 12 fois la normale, de l'ASAT à 10 fois la normale, des phosphatases alcalines à 3 fois la normale, de la gamma-GT à 7 fois la normale et une bilirubinémie à 12 µmol/L. L'activité des LDH était à 2 fois la normale et le taux de prothrombine à 40%. La radiographie pulmonaire et l’électrocardiogramme étaient normaux. Les prélèvements bactériologiques (examen cytobactériologique des urines, hémocultures, prélèvements bactériologiques cutanés) revenaient négatifs. Les sérologies pour hépatite A, B et C, cytomégalovirus, virus d'Epstein-Barr, VIH, rougeole, toxoplasmose, et ASLO n'apportaient pas d'argument pour une infection récente. Le tableau clinique et biologique était donc compatible avec un DRESS syndrome. La carbamazipine et la dépakine étaient arrêtées et remplacées par le sabril associé à l'urbanyl. Devant la progression rapide de l’éruption cutanée et la sévérité de l'atteinte hépatique, la patiente était traitée par une corticothérapie par voie orale à la dose de 1mg/kg/j. L’évolution était favorable en deux semaines avec disparition de la fièvre et des lésions cutanées et des adénopathies. Le bilan hépatique et le taux des éosinophiles étaient normalisés au bout de 5 semaines. Les tests épicutanés réalisés ultérieurement au centre de pharmacovigilance confirment l'allergie isolée au carbamazepine. Aucune récidive n’était observée après un recul de 3 ans.

## Discussion

Le syndrome d'hypersensibilité médicamenteuse ou DRESS syndrome est une toxidermie grave qui peut mettre en jeu le pronostic vital [3]. Ses caractères cliniques et biologiques sont maintenant bien connus et permettent son identification [4]. Son diagnostic repose sur une triade incluant une éruption cutanée, des anomalies hématologiques à type d'hyperéosinophilie ou d'hyperlymphocytose atypique et une atteinte viscérale intéressant notamment le foie et le rein [1, 3]. Plusieurs critères diagnostiques définissant le DRESS syndrome ont été proposés dans la littérature, mais les plus largement répandu sont ceux issus des données du registre européen des effets indésirables cutanés sévères (REGISCAR) ayant établi un score diagnostique distinguant les DRESS avérés, probables, possibles et absents (Tableau 1). Ce score regroupe les signes les plus fréquemment observés au cours des DRESS répertoriés dans la littérature et attribue un score à chaque symptôme clinique, biologique ou chronologique présenté par le patient. Le total, compris entre 0 et 9, permet de déterminer la probabilité du diagnostic de DRESS; le diagnostic étant retenu pour un score supérieur ou égal à 5 [4, 5]. Tous nos patients présentaient un syndrome de DRESS certain (Tableau 2). Toutefois, la sensibilité et la spécificité de ce score ne sont pas encore établies.

**Tableau 1:** Critères du groupe REGISCAR: classement du DRESS comme certain, probable, possible ou exclu [8]

Score	-1	0	1	2	Min	Max
**Fièvre ≥ 38,5 °C**	Non/U	Oui			-1	0
**Polyadénopathies**		Non/U	Oui		0	1
**Éosinophilie**		Non/U			0	2
Éosinophiles			0,7–1,499×109/L	≥ 1,5× 109/L		
Éosinophiles, si leucocytes < 4,0 × 109/L			10-19,9%	≥ 20%		
**Lymphocytes atypiques**		Non/U	Oui		0	1
**Atteinte cutanée**					-2	2
Étendue du rash (% surface corporelle)		Non/U	>50%			
Rash évocateur de DRESS	Non	U	oui			
Biopsie cutanée en faveur du DRESS	Non	Oui/U				
**Atteinte viscérale** ^**a**^					0	2
Foie	Non/U	Oui				
Rein	Non/U	Oui				
Muscle/cæur	Non/U	Oui				
Pancréas	Non/U	Oui				
Autres organes	Non/U	Oui				
**Régression≥ 15 jours**	Non/U	Oui			-1	0
**Évaluation d'autres causes**						
Facteurs antinucléaires						
Hémocultures						
Sérologies pour HAV/HBV/HCV						
Chlamydia/Mycoplasma						
Si non positif ou ≥3 négatif			Oui		0	1
**Score total**					-4	9

U: non connu; HAV: hepatitis A virus; HBV: hepatitis B virus; HCV: hepatitis C virus.

A Après exclusion de tout autre diagnostic: 1, un organe; 2, deux organes ou plus. Score final < 2, exclu; score final 2–3, possible; score final 4–5, probable; score final > 5, certain.

**Tableau 2:** Caractéristiques cliniques, biologiques et évolutives des patients

Observations	1	2	3
Sexe	Masculin	Féminin	Féminin
Age (ans)	37	45	17
Medicaments incriminés	Carbamazipine	Salazopyrine	carbamazipine
Délai (semaines)	8	6	4
Atteinte cutanée	Eruption maculopapuleuse	Eruption maculopapuleuse	Eruption maculopapuleuse
Fièvre ≥ 38,5°C	Oui	Oui	Oui
Oedème facial	Oui	Oui	Oui
Adénopathies	Oui	Oui	Oui
Atteinte hépatique	Cytolyse 8 x normale Cholestase	Cytolyse 8 x normale	Cytolyse 12 x normale Cholestase Insuffisance hépatocellulaire
Anomalies hématologiques	Hyperéosinophilie à 3180 éléments/ mm^3^	Hyperéosinophilie à 2800 éléments/ mm^3^	Hyperéosinophilie à 3600 éléments/ mm^3^ Syndrome mononucléosique
Traitement	Arrêt carbamazépine Prednisone 1 mg/Kg/j	Arrêt carbamazépine Prednisone 1 mg/Kg/j	Arrêt carbamazépine Prednisone 1 mg/Kg/j
Evolution	- Régression du rash cutané -Disparition des adénopathies -normalisation de la fonction hépatique -Pas de récidive	- Régression du rash cutané -Disparition des adénopathies -normalisation de la fonction hépatique -Pas de récidive	- Régression du rash cutané -Disparition des adénopathies -normalisation de la fonction hépatique -Pas de récidive
Recul (ans)	2	2	3
Score	6	6	6

Le DRESS syndrome est une réaction d'hypersensibilité médicamenteuse retardée, survenant deux à six semaines après la première prise du médicament incriminé. Le délai de survenue de cette réaction est donc plus long que celui observé dans la plupart des autres réactions cutanées médicamenteuses graves (syndrome de Lyell notamment), il est donc en grande partie responsable du retard diagnostique fréquent dans ce syndrome [2]. De nombreux médicaments ont été impliqués, notamment des antiépileptiques (phénytoïne, carbamazépine, phénobarbital), l'allopurinol, des antirétroviraux (névirapine), des antibiotiques (minocycline) et des anti-inflammatoires non stéroïdiens (ibuprofène, phénylbutazone) [2, 3]. Pour nos patients, les médicaments incriminés étaient la carbamazipine dans deux cas et la salazopyrine dans un cas. Le délai moyen ente la prise médicamenteuse et la survenue du DRESS syndrome était de six semaines. Le DRESS syndrome associe dans sa forme complète une éruption cutanée maculopapuleuse prurigineuse polymorphe avec un œdème du visage un œdème facial à prédominance orbitaire, une fièvre, des adénopathies périphériques, des atteintes viscérales potentiellement sévères (hépatique, cardiaque, pulmonaire, rénale) ainsi qu'une hyperéosinophilie sanguine et dans 40% des cas un syndrome mononucléosique [6]. Les manifestations dermatologiques sont quasi constantes. Il s′agit initialement d′un exanthème morbilliforme, difficilement distinguable d′une forme bénigne de toxidermie [4]. L′attention doit être attirée par la fièvre, une altération marquée de l′état général et un œdème facial à prédominance péri-orbitaire, présent dans la moitié des cas. L′évolution est marquée par la coalescence et l′infiltration des lésions maculopapuleuses aboutissant à la formation de vastes plaques ou nappes infiltrées oedémateuses voire une érythrodermie, présente chez plus de 50% des malades. La fièvre, présente dans la majorité des cas, peut culminer à 39-40°C notamment dans les formes érythrodermiques. L′état général est altéré. Une polyadénopathie est souvent retrouvée [3, 4, 6].

La sévérité du DRESS est liée aux atteintes viscérales qui sont variées: insuffisance rénale, pneumopathie interstitielle, myocardite, pancréatite, méningo-encéphalite, ou syndrome d'activation macrophagique [2]. L'atteinte multiviscérale distingue le DRESS syndrome des réactions cutanées médicamenteuses communes (toxidermies) et le classe dans la catégorie des réactions cutanées médicamenteuses graves avec une mortalité globale évaluée à 10% par défaillance viscérale liée à une infiltration éosinophilique [6]. L'atteinte hépatique représente l'atteinte viscérale la plus fréquente et la principale cause de mortalité [7, 8]. Une cytolyse est retrouvée dans plus de 80% des cas. Elle peut être intense avec des chiffres de transaminase atteignant 10 à 20 fois la normale. Une cholestase anictérique, avec élévation de l'activité des phosphatases alcalines et de gamma-GT, lui est souvent associée. Une atteinte cholestatique pure avec ictère est plus rare. Une détérioration de la fonction hépatique avec baisse du taux de prothrombine signe une forme grave, parfois mortelle [8]. Chez nos patients, l'atteinte hépatique était constante. Une insuffisance hépatocellulaire était constatée dans un cas. Un traitement précoce a permis une évolution favorable.

Le traitement du DRESS syndrome consiste en l'arrêt immédiat du médicament, qui sera par la suite définitivement contre-indiqué, ainsi que tous les médicaments de la même classe thérapeutique en raison de réactivités croisées entre différents médicaments d'une même classe [2, 3]. La précocité de l'arrêt est indispensable car un retard à l'arrêt est associé à un mauvais pronostic. Dans les formes sévères (transaminases > 5 fois la normale, insuffisance rénale organique, pneumopathie, hémophagocytose, atteinte cardiaque, etc.), une corticothérapie générale (1 mg/kg/j) est nécessaire. Cette corticothérapie sera poursuivie jusqu'au contrôle complet du DRESS avec une décroissance progressive souvent sur plusieurs mois [2, 3, 6]. Les formes mettant en jeu le pronostic viral justifieront d'un traitement par immunoglobulines par voie veineuse (IG IV) en association avec les corticoïdes [2, 3]. Une étude récente a montré que les IG IV seules n’étaient pas adaptées au DRESS [9]. Un traitement antiviral (ganciclovir, cidofovir) pourra être associé à la corticothérapie générale et aux IGIV dès mise en évidence de la réactivation virale [2, 3]. Les DRESS syndromes doivent être déclarés à la pharmacovigilance et le patient sera informé de la nécessité de ne pas reprendre ce médicament. Des tests cutanés à distance aideront à l’étude d'imputabilité [10].

Le DRESS justifie une surveillance prolongée. La surveillance biologique repose sur un contrôle des examens biologiques deux fois par semaine jusqu’à un mois après la normalisation des signes clinico-biologiques, puis une fois par semaine pendant trois mois, voire plus selon l’évolution. Une vigilance particulière s'impose lors de l'introduction de nouveaux traitements. Des réactions croisées sont possibles entre des molécules de nature biochimique non apparentée, illustrant la singularité des mécanismes pathogéniques du DRESS en comparaison des autres toxidermies [2, 3, 6]. La survenue de poussées évolutives à distance n'est pas rare même en absence de toute nouvelle prise médicamenteuse, en particulier, lors de la décroissance de la corticothérapie générale qui doit être progressive. Des manifestations d'auto-immunité (diabète, réaction du type réactions du greffon contre l'hôte, dysthyroïdie, lupus, etc.) peuvent survenir à distance [2, 3].

Chez nos patients, le diagnostic précoce du DRESS syndrome a permis d'arrêter immédiatement le médicament incriminé et d'instaurer une corticothérapie par voie générale devant la présence d'une atteinte hépatique sévère. L’évolution était favorable avec disparation des lésions cutanées, des adénopathies et normalisation du bilan hépatique et du taux des éosinophiles. Aucune récidive n’était observée.

## Conclusion

Le syndrome DRESS demeure une pathologie rare mais grave. Le diagnostic doit être évoqué devant un tableau associant une éruption fébrile et des signes systémiques faisant suite à une prise médicamenteuse. La précocité du diagnostic est fondamentale pour l'arrêt définitif de ou des médicaments suspects. Le traitement n'est pas bien codifié mais repose actuellement sur la corticothérapie générale.
